# Editorial

**Published:** 1897-07

**Authors:** 


### Editorial.

This is an iconoclastic age. Our most cherished traditions are assailed and we begin to be harassed with the doubt that much which we regard as established fact is but illusion. Of course we all know since the discovery of Donelly's cryptogram that Shakspeare was a mere poaching barn-stormer who filched Bacon out of his dramas, and that the episode of John Smith and Pocahontas was but a myth to be classed with the story of Jack the Giant Killer, and that the lowly Nazarene was a rabid fanatic of the type of the Mahdi, whose existence, if indeed he ever did exist, was entirely unimportant. These are all popular fallacies, long since passed out of belief of intelligent, enlightened people. But there were some, even at this late date, who believed that Ephraim McDowell performed the first ovariotomy in 1809, until the great, lonesome Lawson Tait, the Brummagem Jack the Ripper, informed us in the *Buffalo Medical Journal* of June that one Robert Houston deserves the credit, for in 1701 he evacuated by an incision through the abdomen the contents of an ovarian cyst. Since the mighty fiat has gone out from Brummagem that Houston must be the man, we must obediently say our credo. Obediently, but not unmindful of the fact that Tait is the prince of obstructionists, the great I AM of the anti-vivisectionists, the sworn friend of the pyogenic micro-organism, because a disbeliever in the doctrine of antisepsis, and above all a Briton. The gauziness of his argument to support Houston is altogether unworthy of this abdominal Napoleon. It is mere juggling with words. "What is an ovariotomy—and I think the question is quite easy. A surgical operation by which it is thought to cure a tumor of an ovary." Upon this quibble, the claims of McDowell, which have raised monuments to him and made his name luminous in the history of medicine, would be overthrown.

According to this definition Battey's operation is not an ovariotomy because Battey did not think to cure a tumor of an ovary, because there was no tumor. Ovariotomy means cutting into an ovary, or literally cutting an ovary, and is an insufficient word. Oophorectomy means cutting out an ovary, which McDowell did and which Houston did not. Orchidotomy means cutting into a testicle; orchidectomy means cutting out a testicle or both testicles. There may be no distinction between the two operations in the mind of the mighty Tait, but the patient is apt to realize a difference.

But we have no intention of arguing with this Jeroboam, the son of Nebat. We are too small game for his gun. Mr. Greig Smith will take care of him in his response. It is a deplorable tendency of this sophisticated age to seek to tear down and destroy the true and the good, without any other motive than to arouse a feeling of savage joy in witnessing the work of demolition, and maliciously to shake simple faith in men and things.

---

J. Greig Smith the distinguished English surgeon is dead.

---

The consolidation of Bellevue and the University of New York is reported to have fallen through.

---

New York has suffered by death the loss of two of her foremost physicians, J. Lewis Smith and William T. Lusk.

---

The annual report of the Atlanta Board of Health has been issued. It has been very active in the improvement of the city's sanitation during the past year.

---

The second annual meeting of the Southern Railway Surgeons was held at Chattanooga, June 29 and 30. There was a large attendance and many interesting papers were presented.

